# Are Kidneys Affected by SARS-CoV-2 Infection? An Updated Review on COVID-19-Associated AKI

**DOI:** 10.3390/pathogens13040325

**Published:** 2024-04-16

**Authors:** Fabrizio Fabrizi, Luca Nardelli, Anna Regalia, Francesca Zanoni, Giuseppe Castellano

**Affiliations:** 1 Division of Nephrology, Dialysis and Kidney Transplant, Foundation IRCCS Cà Granda Ospedale Maggiore Policlinico, 20122 Milan, Italy; luca.nardelli@policlinico.mi.it (L.N.); anna.regalia@policlinico.mi.it (A.R.); francesca.zanoni@policlinico.mi.it (F.Z.); giuseppe.castellano@policlinico.mi.it (G.C.); 2 Department of Clinical Sciences and Community Health, University School of Medicine, 20122 Milan, Italy

**Keywords:** acute kidney injury, chronic kidney disease, COVID-19, kidney disease, SARS-CoV-2 infection

## Abstract

Background: Human kidneys are an important target of SARS-CoV-2 infection, and many renal abnormalities have been found in patients with SARS-CoV-2 infection, including proteinuria, hematuria, and acute kidney injury. Acute kidney injury is now considered a common complication of COVID-19, and the epidemiology of AKI in SARS-CoV-2-infected patients continues to be controversial. Aim and Methods: We have carried out a narrative review to evaluate the frequency and risk factors for AKI among patients hospitalized due to COVID-19, and the latest surveys on this topic have been included. The mechanisms by which AKI occurs in COVID-19 patients have also been reviewed. Results: Multiple risk factors for the development of AKI in patients with SARS-CoV-2 infection have been identified; these have been classified in various groups (management and background factors, among others). SARS-CoV-2 targets the kidneys by indirect activity, but SARS-CoV-2 infects tubular epithelial cells and podocytes. We retrieved 24 reports (*n* = 502,593 unique patients with SARS-CoV-2 infection) and found an incidence of AKI of 31.8% (range, 0.5% to 56.9%). Only a minority (*n* = 2) of studies had a prospective design. We found that the AKI risk was greater in SARS-CoV-2 patients who underwent in-hospital deaths vs. those who survived; the summary estimate of the unadjusted RR of AKI was 2.63 (95% CI, 2.37; 2.93) (random-effects model). A stratified analysis showed that the incidence of AKI was greater in those reports where the frequency of COVID-19-positive patients having comorbidities (diabetes mellitus, arterial hypertension, and advanced age) was high. The unadjusted relative risk (aRR) of AKI was greater in SARS-CoV-2 patients who underwent ICU admission vs. those who did not; the pooled estimate of AKI risk was 2.64 (95% CI, 1.96; 3.56) according to the random-effects model. Conclusions: AKI is a common complication of hospitalized SARS-CoV-2-infected patients, and some comorbidities are important risk factors for it. The direct activity of the virus on the kidneys has been mentioned in the pathogenesis of AKI in SARS-CoV-2 patients. Further studies are ongoing in order to identify the mechanisms underlying the kidney injury in this population. The role of AKI on survival in SARS-CoV-2-infected patients is another area of active investigation.

## 1. Introduction

Coronaviruses are viruses belonging to the *Coronaviridae* family that can cause severe illnesses, such as Middle East respiratory syndrome (MERS-CoV) and severe acute respiratory syndrome (SARS-CoV). Coronaviruses appear like spiked rings when observed by electron microscope and COVID-19 (also named coronavirus disease 2019) is the illness caused by a virus classified as SARS-CoV-2 (severe acute respiratory syndrome coronavirus-2) [1]. The first human cases of COVID-19 were first reported from Wuhan city, China, in December 2019; since then, COVID-19 has spread rapidly all over the world. The World Health Organization (WHO) declared COVID-19 a pandemic disease in March 2020 [2]. An update from the World Health Organization (10 March 2024) reports that globally, the number of confirmed cases of SARS-CoV-2 is 774,889,074 (cumulative total), including 7,038,023 deaths (cumulative total), reported to the WHO [3]. 

From a clinical standpoint, typical manifestations in patients with SARS-CoV-2 infection include unproductive cough, fatigue, fever, dyspnea, and imaging evidence of pneumonia. The course of infections by SARS-CoV-2 is largely unpredictable, as SARS-CoV-2 ranges from asymptomatic infection to multi-organ systemic failure and death; respiratory failure remains the most important cause of death in patients with COVID-19. It appears that at least a third of people with SARS-CoV-2 infection do not have noticeable symptoms, and the deleterious manifestations of SARS-CoV-2 have been observed in various organs and tissues.

Circumstantial evidence has been recently accumulated, showing that human kidneys are an important target of SARS-CoV-2, and this is in contrast with preliminary data on the SARS-CoV-2-infected population [4,5]. Kidney impairment is common in patients with COVID-19, and SARS-CoV-2 targets the kidneys in various ways. In SARS-CoV-2 infection, the virus’ spike proteins bind to the angiotensin-converting enzyme receptor 2 (ACE2) on the cell surface and are cleaved by membrane proteases, allowing for the virus to fuse with the host cell. Kidney cells are directly infected by ACE2 receptor 2 (ACE2), which is present on the apical brush borders of the proximal tubules as well as parietal epithelial cells, mesangial cells, and podocytes [6]. In addition, kidneys can be indirectly affected by other mechanisms, including ischemia, coagulation alterations, multi-organ injury, abnormalities in the immune system, and others [7].

**Figure 1:**
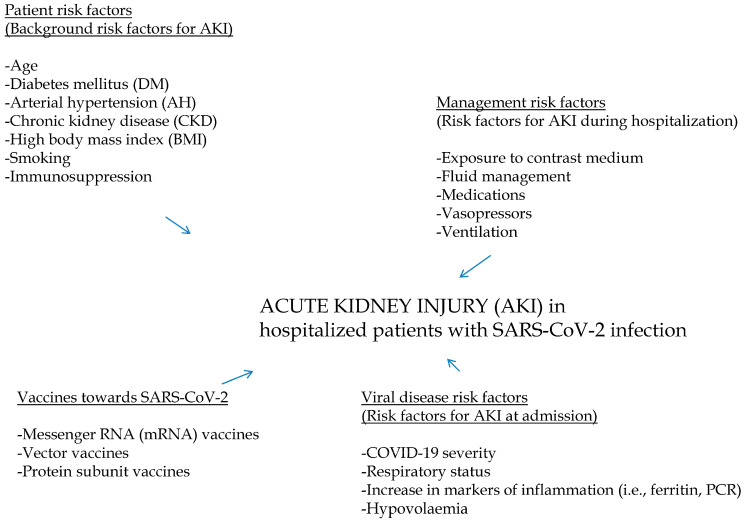
Pathogenesis of AKI in COVID-19-positive patients. Some agents (such as vaccines towards SARS-CoV-2) lower the incidence of AKI in SARS-CoV-2 patients, and others (i.e., factors associated with the viral disease per se, factors linked to SARS-CoV-2 therapy, or background parameters) appear to increase the AKI rate in the same cohort.

Acute kidney injury appears to be a common complication at the kidney level in patients with SARS-CoV-2 infection, and its pathogenesis has clearly multifactorial nature. Various factors play a pivotal role on the risk of AKI in patients with SARS-CoV-2 infection. Some factors (such as vaccines against SARS-CoV-2) reduce the frequency of AKI in SARS-CoV-2-positive patients, while others (factors associated with the viral disease per se or factors linked to the management of SARS-CoV-2 or demographic/clinical parameters) appear to increase the risk of AKI in the same population (Figure 1).

In this article, we review the current knowledge regarding the onset of AKI in patients with COVID-19; the mechanisms underlying the incidence of AKI in this setting have been extensively reviewed.

## 2. SARS-CoV-2 Infection and Kidneys: AKI

The incidence of AKI in patients with COVID-19 has been reported in the medical literature to a variable extent [8]. AKI is uncommon in patients with mild COVID-19 and is frequent in patients with COVID-19 during their hospital stay; the frequency of AKI is even greater in those patients with severe COVID-19. Other factors help to explain the wide variability in the incidence of AKI. Research during SARS-CoV-2 infection has been hampered by the pressure to release data quickly; variable quality in study designs or conduct has been noted, as the occurrence of selection bias, information or collider bias, or confounding has already been highlighted [9]. Most studies but not all have adopted the Kidney Disease Improving Global Outcomes (KDIGO) consensus definition of AKI [10]. The frequency of AKI in patients with COVID-19 has been reported greater than historical controls, but this has been explained by some authorities, as SARS-CoV-2 infection could result in a more severe disease in comparison with non-COVID hospitalized patients (for community-acquired pneumonia or other respiratory viruses). AKI occurs frequently in community-acquired pneumonia (49%, 21%, and 30% developing stage 1, 2, and 3 AKI, respectively [11].

A novel systematic review and meta-analysis has been made by our group. Thirty-nine clinical studies have been retrieved (*n* = 25,566 unique patients), and the pooled incidence of AKI was 0.154 (95% CI, 0.107; 0.201, *p* < 0.0001) across the reports; in other words, the frequency of AKI in hospitalized patients with SARS-CoV-2 was 15.4%. However, significant and obvious heterogeneity was found (*p*-value from *Q* test, 0.0001, *I*^2^ = 99.4%) [8]. We found large variability in the frequency of AKI among the papers enrolled in the review; this could be related to various factors, such as the severity of illness by SARS-CoV-2 (as reported above), patient characteristics (i.e., age), or differences in daily clinical practice during their hospital stay. Stratified analyses were undertaken to explain the heterogeneity across studies, and some of these were not meaningful. The analysis by the fixed-effects model yielded similar findings to the random-effects model. The summary estimate for the frequency of COVID-19-positive patients who underwent RRT due to AKI during their hospital stay was 0.043 (95% CI, 0.031; 0.055) [8].

The pooled estimate of AKI incidence in patients with severe COVID-19 was 0.53 (95% CI, 0.427; 0.633); again, there was consistent and obvious heterogeneity (*p*-value from *Q* test, 0.0001, *I*^2^ = 97.26%). The pooled OR of AKI incidence among deceased COVID-19-positive patients was larger than among survivors, 15.4 (95% CI, 20.99–11.4). A few investigators assessed the relationship between AKI and death risk with a multivariate analysis (*n* = 5, *n* = 5435 unique patients). The association between AKI and mortality remained significant in many comparisons; the summary estimate for adjusted relative risk (aRR) with the Cox proportional hazard model of all-cause mortality among hospitalized patients with COVID-19 and AKI was 2.8 (95% CI, 0.96; 8.13) according to the random-effects model [8].

## 3. SARS-CoV-2 and Kidneys: Risk Factors for AKI

Risk factors for AKI occurrence in patients with SARS-CoV-2 infection have been categorized in various groups, including pre-existing comorbidities (1), biochemical or clinical parameters at hospital admission (2), and abnormalities in the management of AKI during hospitalization (3). The first group includes diabetes, arterial hypertension, aging, male gender, weight overload, and chronic kidney disease (Figure 1). In particular, CKD has recently been re-evaluated as an important risk factor for AKI.

In the context of our systematic review and meta-analysis of observational studies [8], we had carried out a meta-regression analysis to identify the potential association between the impact of many continuous covariates (age, arterial hypertension, diabetes, etc.) on the outcome of interest (AKI incidence among hospitalized COVID-19 patients). Age (*p* < 0.007) and arterial hypertension (*p* < 0.001) were associated with the occurrence of AKI [8]. It remains unclear whether a relationship between AKI and race exist; some papers have reported a greater incidence of AKI among Afro-American patients even after adjustment for several confounders, and genetic factors have been cited to explain it (patients with the APOL1 allele have a greater AKI incidence) [12].

In the second group, many clinical parameters largely related to the severity of COVID-19, such as great respiratory frequency (>20), or blood biochemistries, including inflammatory markers (IL-6, C-reactive proteins, fibrinogen, etc.), support AKI incidence. An unsatisfactory fluid management at either extreme, exposure to nephrotoxic agents, and mechanical ventilation are well-known risk factors for AKI (third group). Differences in daily clinical practice (ventilation choices and medications, among others) could be taken into account.

## 4. SARS-CoV-2 and Kidneys: AKI Pathogenesis and Direct Renal Tropism

**Figure 2:**
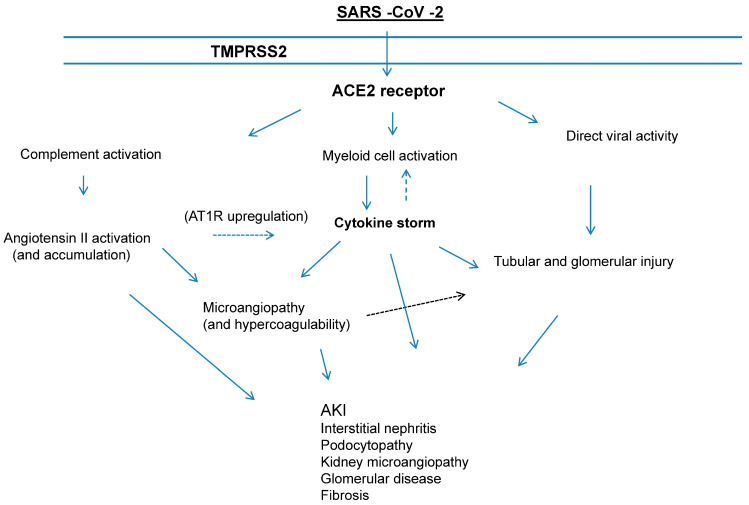
Molecular mechanisms of AKI in patients with SARS-CoV-2 infection.

Multiple factors can contribute to the pathogenesis of AKI in patients with COVID-19; the direct activity of SARS-CoV-2 on the kidney parenchymal cells has been mentioned by several authors. The presence of viral particles in tubular epithelial cells, podocytes, and urine on electron microscopy has been already recorded [13]. The infectious period of SARS-CoV-2 is rather short, and this hampers the identification of the virus in renal tissues, as kidney biopsies or autopsy investigations have been made often days to weeks after the development of SARS-CoV-2-associated AKI. The structure of SARS-CoV-2 includes large proteins with a high molecular weight (around 500 kDa), and this prevents it from filtering through the glomerular barrier into intact kidneys [14]. Thus, some authors state that ACE2 expression seems to be the pivotal mechanism of infectivity (Figure 2). 

## 5. SARS-CoV-2 and Kidneys: AKI Pathogenesis and ‘Cytokine Storm’

COVID-19 has a broad clinical spectrum; the lungs are the most important organs affected in the disease, but the involvement of other organs, such as the gastrointestinal tract, bone marrow, liver, heart, kidneys, and others, has been observed. The multi-organ involvement in patients with SARS-CoV-2 infection is at least in part explained by the wide distribution of ACE2, that is, the molecular receptor that allows for host cell infection by SARS-CoV-2. After the SARS epidemic in 2003, ACE2 was recognized as a viral receptor [15]. Some authors initially mentioned that ACE2 was entirely and exclusively expressed in the lungs (pneumocytes I and II), heart, kidneys, and testes. After that, ACE2 expression was also observed in adipocytes, brain, gastrointestinal tract, and nasal and oral mucosa [16]. However, the clinical and physiological significance of ACE2 expression in most of these tissues is still unclear.

The pathogenesis of AKI in COVID-19 patients has numerous reasons, such as impaired kidney perfusion and exposure to nephrotoxic agents. In addition to the direct cytopathic effects of the virus, indirect ways have been cited. The lung involvement in COVID-19 patients gives the reduced oxygen saturation of the blood and can support an ischemic damage at the kidney level. Also, a potential role of a ‘cytokine storm’ has been suspected; a ‘cytokine storm’ is an acute hyper-inflammation response that may be the cause of critical illness in many clinical conditions, such as cancer, sepsis, or viral infections. Iwasaki and colleagues [17] suggested that once ACE2 receptors are linked to SARS-CoV-2, angiotensin II accumulates and promotes the activation of angiotensin type I receptor (ATIR) and the reduction of angiotensin synthesis; such events lead to the occurrence of the ‘cytokine storm’. The ‘cytokine storm’ has been cited in critically ill patients with SARS-CoV-2 infection where an uncontrolled systemic inflammatory response leads to kidney failure. The occurrence of the ‘cytokine storm’ has been recorded since the first reports of COVID-19 [18]. The incidence of AKI is quite variable, but its frequency is greater in critically ill patients; patients with SARS-CoV-2 infection who undergo hospitalization in ICUs have greater levels of IL-6, IL-8, TNF-α, IL-1β, and interferon-γ in comparison with non-critically ill patients [18]. Such a phenomenon suggests a potential role of the ‘cytokine storm’ where pro-inflammatory IL-6, TNF-α, and IFN-γ are considered the most important causative cytokines. The ‘cytokine storm’ produces intrarenal inflammation, increased vascular permeability, and volume depletion, resulting in AKI. TNF-α and IFN-γ are greatly upregulated in the serum of patients with severe COVID-19 and generates apoptosis or necroptosis [19]. Cytokines are strongly related to cell death mechanisms and are involved in a positive feedback process.

ACE and TMPRSS2 are expressed in proximal convoluted tubule cells, proximal straight tubule cells, and podocytes. SAS-CoV-2 enters glomerular capillaries through the blood circulation and then attacks podocytes or enters kidney tubular fluid to contact receptors in proximal tubules. This results in podocyte impairment, tubular damage, endothelial dysfunction, and collapsing glomerular disease (Figure 2).

## 6. SARS-CoV-2 and Kidneys: AKI and Compromised Immune Response

Some authors have found a virus-induced activation of the complement system [19]; one study on renal biopsies from six patients with severe COVID-19 found extensive complement deposition on kidney tubules, and this could support the hypothesis that complement deposition plays a pivotal role in kidney injury [20]. The complement system has a double role in patients with SARS-CoV-2 infection; it is involved in the immune response against SARS-CoV-2 but also in the pathogenesis of COVID-19-associated tissue inflammation. In fact, the complement system appears to work like a double-edged sword in patients with severe viral infections; it gives the first immune response against SARS-CoV-2 infection but, on the other side, supports inflammation, tissue injury, and coagulation abnormalities. In patients with severe COVID-19, the ‘cytokine storm’ probably plays a role in the development of some manifestations, including acute respiratory distress syndrome, thromboembolic diseases (such as acute ischemic stroke), acute kidney injury, or vasculitis.

An important role of the dysregulated immune response to SARS-CoV-2 infection in COVID-19 patients has been recently suggested. Various types of immunity exist: innate immunity (rapid response) and adaptive immunity (slow response); in addition, passive immunity occurs, including natural and artificial immunity (received from the maternal side and vaccine/medications, respectively) [21]. After viral infection, the immune response is made by B cells (assisted by T cells), which differentiate into plasma cells with the aim to produce an antibody specific to a viral antigen. Neutralizing the antibody blocks the virus from entering into host cells and limits the infection. An IgM autoantibody response against ACE2 has been reported; purified anti-ACE2 IgM can stimulate complement components in endothelial cells [22]. Of note, anti-ACE2 IgM synthesis has been linked to a consistent anti-SARS-CoV-2 spike protein IgG response. The conclusion of the authors was the occurrence of an anti-idiotype IgM response cross-reacting with ACE2 [22].

The cellular immunity response develops inside infected cells, and this is mediated by T cells. The adaptive immune response is usually driven by T helper cells, and cytotoxic T cells play a pivotal role in the clearance of viral-infected cells [23]. Novel data reported a great incidence (about 40%) of lymphopenia in patients with COVID-19 [23], and lymphopenia portends poor prognosis [24]. Liu and coworkers found an inverse correlation between T-cell counts and the kinetics of cytokine levels in patients with severe COVID-19 [25]. Acute lung injury increases systemic levels of cytokines and helps the release of damaged-associated molecular patterns (DAMPs) from injured cells. According to Land W. [26], the trajectory from a localized immune response to a systemic immune and inflammatory response (leading to the ‘cytokine storm’) is supported by the development of DAMPS.

## 7. SARS-CoV-2 and Kidneys: Epidemiology of AKI (Pooled Analysis)

In the context of the current narrative review, we have carried out a pooled analysis of the medical literature regarding the incidence of AKI in hospitalized patients with COVID-19. We have included 24 reports (*n* = 502, 593 unique patients) and found an incidence of AKI of 31.8% (range, 0.5% to 56.9%) (Table 1) [27,28,29,30,31,32,33,34,35,36,37,38,39,40,41,42,43,44,45,46,47,48,49,50]. Many surveys (*n* = 9) were published very recently (2022–2023) (Table 1). As listed in Supplementary Table S1, most reports had a retrospective design, and a few (*n* = 2) had a prospective nature [35,49]. Some (*n* = 7) had a multicenter approach [31,38,40,42,45,46,50]. The weighted average of male frequency was 52% (range, 37.1% to 74.6%). The weighted average of chronological age was 61.7 years. The average of the arterial hypertension rate was 46.6% (range, 15% to 72.4%) (Table 1). The rate of diabetics was between 7.4% and 46.7% (Table 2), and the average of diabetic frequency was 24.5%.

A stratified analysis (Table 3) reported a greater incidence of AKI in reports where the rate of DM, arterial hypertension, and aging among COVID-19-positive was large. The remaining comparisons were not meaningful.

**Figure 3:**
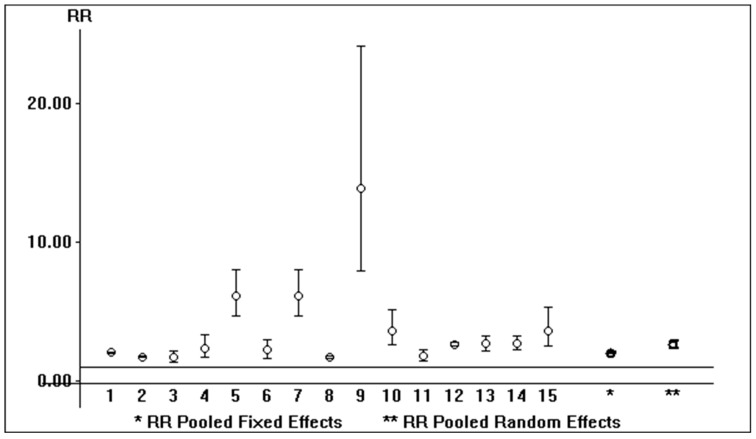
Relative risk of AKI in hospitalized patients with SARS-CoV-2 infection who underwent in-hospital mortality vs. those who survived (*n* = 15 studies) (fixed- and random-effects model).

The link between in-hospital mortality and AKI risk was also addressed (*n* = 15 papers). We found a relationship between in-hospital mortality and AKI risk, and the summary estimate of the unadjusted RR of AKI in COVID-19 patients undergoing in-hospital mortality vs. those who survived was 2.63 (95% CI, 2.37; 2.93) according to a random-effects model (Figure 3). The fixed model yielded identical results. 

**Figure 4:**
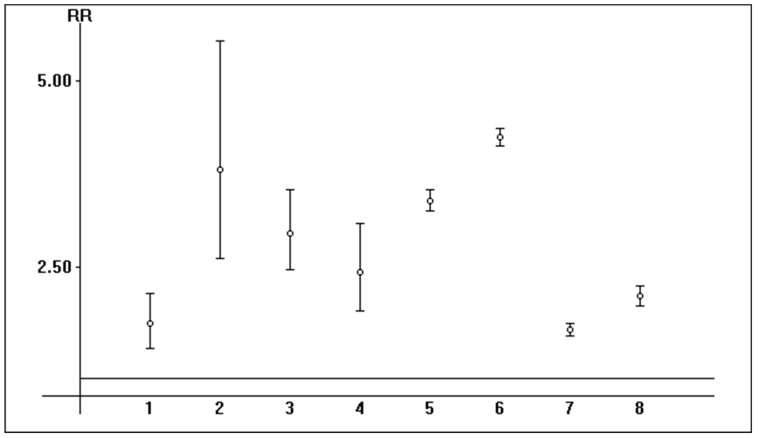
Relative risk of AKI in hospitalized patients with SARS-CoV-2 infection who underwent ICU admission vs. those who did not (*n* = 8 studies) (fixed- and random-effects model) (Odd man out).

The relationship between AKI risk and ICU admission was detailed in some (*n* = 8) reports, and the incidence of AKI was greater in those who underwent ICU hospitalization. The pooled estimate of the unadjusted RR of AKI in SARS-CoV-2 patients who underwent ICU admission vs. those who did not was 2.63 (95% CI, 1.94; 3.58) (the random-effects model) (Figure 4).

## 8. Kidney Disease and Long COVID

Some reports about the kidney outcomes after COVID-19 have been published, but these are biased because of a short duration of follow-up [51]. It has been recently calculated that 1 to 12% of the hundreds of millions infected worldwide with SARS-CoV-2 may experience a protracted course of illness called long COVID [51]. Although long COVID occurs even in asymptomatic patients, cognitive impairment and fatigue are the most common clinical manifestations among these patients; in addition, a growing concern for kidney function decline in the long COVID population exists. It has been discussed that the biochemistry of kidney tubular injury plays a pivotal role in the pathogenesis of long COVID [52]. Recently, a multicenter cohort study (*n* = 9624 patients) concluded that survivors of hospitalization with AKI-associated COVID-19 experienced a 33% lower risk of major adverse kidney events and 69% lower risk of mortality in comparison with AKI related to influenza or other illnesses [53]. Patients were followed up for a maximum of 2 years after hospital discharge. Of note, residual unmeasured confounders contributing to the longitudinal trajectory of kidney function could not be excluded. Studies concerning the kidney function or other markers of kidney dysfunction (i.e., hematuria, proteinuria) in additional cohorts of long COVID are under way.

## 9. Conclusions

SARS-CoV-2 frequently targets the kidneys of hospitalized COVID-19 patients, and the occurrence of AKI was 22% according to our pooled analysis. Multiple risk factors for the onset of AKI have been cited in the literature, and our stratified analysis reported that diabetes mellitus, arterial hypertension, and aging were consistently related to AKI incidence. The impact of AKI on the survival of COVID-positive patients who undergo hospitalization is well known, as has already been observed in numerous clinical investigations and meta-analyses. It appears that SARS-CoV-2 targets human kidneys in direct and indirect ways; numerous clinical trials aimed to obtain a better understanding and management of AKI in COVID-19 patients are underway.

## Figures and Tables

**Table 1:** Acute kidney injury (AKI) in hospitalized patients with SARS-CoV-2 infection: patient characteristics.

Authors	Country	Sample Size, *n*	Male, *n*	Age, Years	Reference Year	Hypertension, *n*
Xu S, et al. [27]	China	355	193 (54.4%)	53.1	2020	125 (35.2%)
Li X, et al. [28]	China	548	279 (50.9%)	60 (48; 69)	2020	166 (30.3%)
Li Q, et al. [29]	China	325	167 (51.4%)	51 (36; 64)	2020	78 (24%)
Pei G, et al. [30]	China	333	182 (54.7%)	56.3 ± 13.4	2020	107 (32.2%)
Fisher M, et al. [31]	US	3345	1776 (53.1%)	64.4 ± 16.4	2020	NA
Argenziano M, et al. [32]	US	1000	596 (59.6%)	63 (50; 75)	2020	601 (60.1%)
Suleyman G, et al. [33]	US	463	204 (44%)	57.5 ± 16.8	2020	295 (63.7%)
Kolhe N, et al. [34]	UK	1161	657 (56.6%)	72.1 ± 15.9	2020	NA
Guan W, et al. [35]	China	1099	637 (57.9%)	47 (35; 58)	2020	165 (15%)
Cheng Y, et al. [36]	China	701	367 (52.3%)	63 (50; 71)	2020	233 (33.4%)
Zahid U, et al. [37]	US	469	268 (57.1%)	64.2 ± 15.5	2020	323 (68.9%)
Chan L, et al. [38]	US	3993	2289 (57.3%)	64 (56; 78)	2021	1527 (38.2%)
Xu H, et al. [39]	Sweden	316	152 (48.0%)	82.2 ± 8.5	2021	158 (50%)
Ng J, et al. [40]	US	9657	5747 (59.5%)	65.1 (51; 81)	2021	5730 (59.3%)
Gameiro J, et al. [41]	Portugal	192	100 (52.1%)	72.2 ± 16.4	2021	131 (68.2%)
Walendy V, et al. [42]	Germany	154,170	79,781 (51.8%)	NA	2022	54,422 (35.3%)
Morieri M, et al. [43]	Italy/Russia	939	497 (52.9%)	62 ± 15.5	2022	511 (54.5%)
Fabrizi F, et al. [44]	Italy	387	247 (63.8%)	66 ± 15.8	2022	174 (44.9%)
Yoo Y, et al. [45]	US	306,061	159,373 (52%)	61.7 ± 18.8	2022	120,353 (51.8%)
Tan B, et al. [46]	International	12,891	9611 (74.6%)	NA	2023	6601 (51.2%)
Kim S, et al. [47]	Korea	858	318 (37.1%)	55.6 ± 20.2	2023	288 (33.6%)
Shchepalina A, et al. [48]	Russia	500	231 (46.2%)	73 (63; 80)	2023	362 (72.4%)
Palomba H, et al. [49]	Brazil	1131	678 (59.9%)	52 ± 15.8	2023	722 (63.8%)
McNicholas B, et al. [50]	International	1699	1112 (65.5%)	56.8	2023	799 (47.0%)

NA = not available.

**Table 2:** Incidence of AKI in hospitalized patients with SARS-CoV-2 infection.

Authors	Caucasian, *n*	CKD, *n*	Diabetes, *n*	AKI, *n*	ICU Admitted, *n*
Xu S, et al. [27]	NA	NA	147 (41.4%)	56 (15.7%)	NA
Li X, et al. [28]	NA	10 (1.8%)	83 (15.1%)	95 (17.3%)	NA
Li Q, et al. [29]	NA	4 (1.2%)	30 (9.2%)	19 (5.8%)	NA
Pei G, et al. [30]	NA	0	76 (22.9%)	35 (10.5%)	NA
Fisher M, et al. [31]	275 (8.2%)	409 (12.2%)	906 (27.1%)	1903 (56.9%)	438 (13.1%)
Argenziano M, et al. [32]	144 (14%)	137 (13.7%)	372 (37.2%)	288 (33.9%)	236 (23.6%)
Suleyman G, et al. [33]	NA	182 (39.3%)	178 (38.4%)	159 (34.3%)	141 (30.4%)
Kolhe N, et al. [34]	876 (75.4%)	224 (19.3%)	255 (21.9%)	304 (26.2%)	96 (8.2%)
Guan W, et al. [35]	NA	8 (0.7%)	81 (7.4%)	6 (0.5%)	55 (5.0%)
Cheng Y, et al. [36]	NA	14 (2%)	100 (14.3%)	36 (5.1%)	73 (10.4%)
Zahid U, et al. [37]	7 (1.5%)	NA	219 (46.7%)	128 (27.3%)	NA
Chan L, et al. [38]	954 (23.9%)	420 (10.5%)	1019 (26%)	1835 (46%)	976 (24.4%)
Xu H, et al. [39]	NA	NA	144 (45.5%)	92 (29.1%)	NA
Ng J, et al. [40]	3328 (34.4%)	492 (5.1%)	3469 (35.9%)	3854 (39.9%)	2409 (24.9%)
Gameiro J, et al. [41]	174 (90.6%)	38 (19.8%)	54 (28.1%)	106 (55.2%)	38 (19.8%)
Walendy V, et al. [42]	NA	23,380 (15.2%)	20,504 (13.2%)	16,773 (10.9%)	29,329 (19%)
Morieri M, et al. [43]	NA	194 (20.7%)	292 (31.1%)	140 (14.9%)	NA
Fabrizi F, et al. [44]	387 (100%)	40 (10.3%)	65 (16.8%)	119 (30.7%)	0
Yoo Y, et al. [45]	192,482 (62.9%)	23,380 (15.2%) NA	70,494 (30.3%)	126,478 (41%)	NA
Tan B, et al. [46]	6215 (48.2%)	4938 (38.3%)	NA	6505 (50.4%)	NA
Kim S, et al. [47]	NA	37 (4.3%)	142 (16.5%)	270 (31.5%)	40 (4.7%)
Shchepalina A, et al. [48]	NA	117 (23.4%)	124 (24.8%)	190 (38%)	76 (15.2)
Palomba H, et al. [49]	NA	33 (2.9%)	269 (23.8%)	376 (33.2%)	1131 (100%)
McNicholas B, et al. [50]	NA	0	509 (30.8%)	355 (20.9%)	1699 (100%)

NA = not available.

**Table 3:** Incidence of AKI in hospitalized patients with SARS-CoV-2 infection (stratified analysis).

Subgroup	Pooled AKI Risk
DM rate > 40%(*n* = 3)	25% (23%; 28%)
DM rate < 15% (*n* = 5)	11% (11%; 12%)
AH rate > 60% (*n* = 6)	36% (34%; 38%)
AH rate < 25% (*n* = 2)	3% (2%; 4%)
Age > 60 yrs (*n* = 14)	41% (41%; 41%)
Age < 60 yrs (*n* = 8)	28% (26%; 29%)
CKD rate > 20% (*n* = 4)	49% (48%; 50%)
CKD rate < 10% (*n* = 8)	37% (36%; 38%)
Reference year (2000) (*n* = 11)	46% (45%; 47%)
Reference year (2003) (*n* = 5)	33% (33%; 35%)
Male rate > 50% (*n* = 5)	33% (32%; 37%)
Male rate < 50% (*n* = 5)	33% (33%; 33%)

## Supplementary Materials

The following supporting information can be downloaded at: https://www.mdpi.com/article/10.3390/pathogens13040325/s1, Table S1: Acute kidney injury (AKI) in hospitalized patients with SARS-CoV-2 infection: study and type design of reports.

## Abbreviations

ACEIs	Angiotensin converting enzymes
AE	Adverse events
AH	Arterial hypertension
AKI	Acute kidney injury
ARBs	Angiotensin receptor blockers
CI	Confidence intervals
CKD	Chronic kidney disease
COVID-19	Coronavirus disease 2019
COPD	Chronic obstructive pulmonary disease
CPAP	Continuous positive airway pressure
DM	Diabetes mellitus
eGFR	Estimated glomerular filtration rate
ESRD	End-stage renal disease
FANS	Non-steroidal anti-inflammatory drugs
HD	Hemodialysis
HFNC	High flow nasal cannula
ICU	Intensive care unit
KDIGO	Kidney Disease: Improving Global Outcomes
NA	Not available
RAAS	Renin–angiotensin–aldosterone system
RRT	Renal replacement therapy
SARS-CoV-2	Severe acute respiratory syndrome coronavirus 2
UTI	Urinary tract infection
VM	Venturi mask
WHO	World Health Organization
