# Supplementary material for: Are Kidneys Affected by SARS-CoV-2 Infection? An Updated Review on COVID-19-Associated AKI
Source: Pathogens. 2024 Apr 16;13(4):325. doi: 10.3390/pathogens13040325 (PMC11054118; doi:10.3390/pathogens13040325)
Supplement: Supplementary file 1 [file pathogens-13-00325-s001.zip › pathogens-2935361-supplementary.pdf]

Supplementary Table S1. Acute kidney injury (AKI) in hospitalized patients with SARS-CoV-2 infection: study and type design of reports.

Authors	Study design and country
**Xu S, et al.**	Retrospective study of patient admitted to two hospitals (from Hubei and Anhui provinces, China)
**Li X, et al.**	Retrospective survey of patients admitted to Tongji Hospital (Wuhan, China)
**Li Q, et al.**	Single-center survey on patients hospitalized in Shangai Public Health Clinical Center, China
**Pei G, et al.**	Retrospective, single-center review from electronic health records (Wuhan, China)
**Fisher M, et al.**	Retrospective, observational study at MHS (Montefiore Health System (Bronx, NYC, NY, US)
**Argenziano M, et al.**	Retrospective manual medical record review from NewYork-Presbyterian/Columbia University Irving Medical Center, New York City (US)
**Suleyman G, et al.**	Case series of consecutive patients with COVID-19 evaluated at Henry Ford Health System in metropolitan Detroit, Michigan (US)
**Kolhe N, et al.**	Investigator-initiated, multicenter, retrospective cohort study from Research and Development Department of University Hospitals of Derby and Burton (UHDB) National Health Service (NHS) Trust (UK)
**Guan W, et al.**	Updated analysis of cases throughout mainland China (552 hospitals)
**Cheng Y, et al.**	Prospective, cohort study of patients with COVID-19 admitted in a tertiary teaching hospital that also encompassed three affiliates following a major outbreak (2020) in Wuhan (China)
**Zahid U, et al.**	Single-center retrospective cohort study (Brookdale, NY) (US)
**Chan L, et al.**	Retrospective, observational review from electronic health records (Mount Sinai COVID Informatics Center, MSCIC) (New York City, NY, US)
**Xu H, et al.**	Retrospective, observational study at 2 geriatric clinics (Stockholm, Sweden)
**Ng J, et al.**	Retrospective, cohort study, from 13 hospitals from electronic health records (All-scripts) (metropolitan New York) (US)
**Gameiro J, et al.**	Retrospective, single-center analysis (Lisbon, Portugal)
**Walendy V, et al.**	Nationwide, retrospective, population-based data on hospitalizations (with COVID-19 and AKI) in Germany
**Morieri M, et al.**	International, multicenter, retrospective study (Padua, Italy and Moscow, Russia)
**Fabrizi F, et al.**	Single-center, retrospective survey (Milano, Italy)
**Yoo Y, et al.**	Retrospective analysis of patients from the National COVID Cohort Collaborative (N3C) (US)
**Tan B, et al.**	Multicenter, observational cohort study from the Consortium for Clinical Characterization of COVID-19 by EHR (electronic health record) (4CE) (US, Spain, Italy, France, Germany, Singapore)
**Kim S, et al.**	Single-center, retrospective, observational study (Seoul and Pyeongtaek, Republic of Korea)

**Shchepalina A, et al.**	Single-center, (retrospective), observational study (Tareev Clinic, Moscow, Russia)
**Palomba H, et al.**	Multi-center, retrospective, observational study (23 private hospitals, Brazil)
**McNicholas B, et al.**	Multi-center, prospective, observational survey (CCCC and LUNG-SAFE studies)
